# Novel Ensemble Approach of Deep Learning Neural Network (DLNN) Model and Particle Swarm Optimization (PSO) Algorithm for Prediction of Gully Erosion Susceptibility

**DOI:** 10.3390/s20195609

**Published:** 2020-09-30

**Authors:** Shahab S. Band, Saeid Janizadeh, Subodh Chandra Pal, Asish Saha, Rabin Chakrabortty, Manouchehr Shokri, Amirhosein Mosavi

**Affiliations:** 1 Institute of Research and Development, Duy Tan University, Da Nang 550000, Vietnam; 2 Future Technology Research Center, National Yunlin University of Science and Technology, 123 University Road, Section 3, Douliou, Yunlin 64002, Taiwan; 3 Department of Watershed Management Engineering and Sciences, Faculty in Natural Resources and Marine Science, Tarbiat Modares University, 14115-111 Tehran, Iran; janizadehsaeid@modares.ac.ir; 4 Department of Geography, The University of Burdwan, West Bengal, Burdwan 713104, India; scpal@geo.buruniv.ac.in (S.C.P.); asishsaha01@gmail.com (A.S.); rabingeo8@gmail.com (R.C.); 5 Institute of Structural Mechanics, Bauhaus Universität Weimar, 99423 Weimar, Germany; manouchehr.shokri@uni-weimar.de; 6 Environmental Quality, Atmospheric Science and Climate Change Research Group, Ton Duc ThangUniversity, Ho Chi Minh City 700000, Vietnam; amirhosein.mosavi@tdtu.edu.vn; 7 Faculty of Environment and Labour Safety, Ton Duc Thang University, Ho Chi Minh City 700000, Vietnam

**Keywords:** gully erosion susceptibility, deep learning neural network, DLNN, particle swarm optimization, PSO, geohazard, geoinformatics, ensemble model, erosion, hazard map, spatial model, deep learning, natural hazard, extreme events

## Abstract

This study aims to evaluate a new approach in modeling gully erosion susceptibility (GES) based on a deep learning neural network (DLNN) model and an ensemble particle swarm optimization (PSO) algorithm with DLNN (PSO-DLNN), comparing these approaches with common artificial neural network (ANN) and support vector machine (SVM) models in Shirahan watershed, Iran. For this purpose, 13 independent variables affecting GES in the study area, namely, altitude, slope, aspect, plan curvature, profile curvature, drainage density, distance from a river, land use, soil, lithology, rainfall, stream power index (SPI), and topographic wetness index (TWI), were prepared. A total of 132 gully erosion locations were identified during field visits. To implement the proposed model, the dataset was divided into the two categories of training (70%) and testing (30%). The results indicate that the area under the curve (AUC) value from receiver operating characteristic (ROC) considering the testing datasets of PSO-DLNN is 0.89, which indicates superb accuracy. The rest of the models are associated with optimal accuracy and have similar results to the PSO-DLNN model; the AUC values from ROC of DLNN, SVM, and ANN for the testing datasets are 0.87, 0.85, and 0.84, respectively. The efficiency of the proposed model in terms of prediction of GES was increased. Therefore, it can be concluded that the DLNN model and its ensemble with the PSO algorithm can be used as a novel and practical method to predict gully erosion susceptibility, which can help planners and managers to manage and reduce the risk of this phenomenon.

## 1. Introduction

Biodiversity in a given area depends on, to a large extent, and supports the most vital natural resources in the soil, which also contribute to the provision of basic human needs such as food, fresh air, and clean water [1]. Therefore, human survival largely depends on the soil component. Soil erosion in the form of gully erosion is a serious global problem, and it continues to pose a threat to soil and water resources, particularly in arid and semi-arid regions of Iran [2,3]. Among the several types of water-induced erosion, gully erosion is a more intense form of soil erosion [4] and is one of the most complex geomorphic phenomena on the Earth’s surface [5]. Such erosional activities also change the shape of the Earth’s landform and produce a rugged topography, which is not suitable for production activities, construction of communication networks, etc. Thus, water-induced soil erosion is the main cause of the destruction of agricultural land, vegetation, and ecosystems, and is ultimately responsible for a devastating land degradation phenomenon. It has been estimated that the annual rate of global soil erosion is approximately 75 billion tons [6]. From an international perspective, Iran ranks second in terms of land losses, and the annual rate of soil erosion is close to 2 to 2.5 billion tons [7]. It has also been predicted that Iran’s average soil erosion rate is 30–32 tons/ha/year, which is 4.3 times the world average (Food and Agriculture Organization of the United Nations (FAO), 1984). In Iran, soil erosion has been estimated to have caused more than USD 1 billion in economic losses (FAO, 2015) and is a national threat [8]. Thus, it is necessary to protect the soil from erosion and to avoid the phenomenon of land degradation worldwide. The main cause of intensive water-related gully erosion and its development is a long hot/dry season followed by an extremely wet period. Therefore, extreme rainfall causes a large amount of surface runoff over the infiltration capacity and easily transports loose soil particles onto the downward slope. Thus, soil erosion related to water in Iran is a major barrier to sustainable development in the areas of agriculture, watershed management, and other activities related to resource development [9]. Hence, the preparation of a gully erosion susceptibility (GES) map is essential for sustainable management, development, and protection of the most vital natural resource on the Earth’s surface, i.e., soil, from intense gully formation and development.

Before preparing a GES map, it is necessary to understand the definition of a gully, its morphological characteristics, causes of occurrences, conditioning factors, and its ultimate impact on the land surface. A gully can be defined as a deep, narrow channel with a depth of more than 30 cm, usually produced by surface and subsurface runoff after a heavy downpour with a temporary flow of water within that channel [8]. Gullies generally transport a large amount of sediment from the high slope or plateau of the unprotected soil surface, i.e., areas with less vegetation, to the down-slope areas of a watershed. It is also a fact that within 5% of the area of a watershed, between 10% and 94% of sediment moves downwards due to gully erosion [10]. According to Poesen [11], different factors affect gully erosion, and these factors are classified into two categories: (a) anthropogenic activities such as excessive use of farm land, overgrazing, unplanned manner of road construction, deforestation, etc., and (b) physical conditions such as topography, climate, vegetation cover, mineral composition in the soil, etc. Depending upon the depth, gullies are classified into three types, i.e., if the depth is <0.3 m then it is called a grove, if the depth is between 0.3 and 2 m it is called a shallow gully, and if the depth is >2 m it is known as a deep gully [12]. Intensive gully erosion causes many environmental problems, such as accumulation of sediment in rivers and devastating floods, as it removes fertile soils, which has a serious impact on agricultural fields, minimizes soil water storage capacity, destroys roads, and ultimately produces badlands [13,14,15]. It is also a well-known fact that similar factors are not responsible for the occurrence of gullies in several places in the world. Gullies are generally formed and developed based on the local topographical, climatological, and hydrological characteristics. Therefore, different gully-prone areas and associated factors need to be identified by mapping the gully erosion susceptibility. Not only this, but a suitable prediction model along with the identification of respective favorable gully erosion conditioning factors (GECFs) are also essential for an unbiased prediction result. Several methods such as statistical, machine learning (ML), and ensemble algorithms have been used for mapping GES, with the combination of remote sensing and geographic information systems. Thus, GES mapping, using the aforementioned newly developed methods, can help land use planners to maintain soil and water resources sustainably and accurately. Furthermore, the potential of the respective region will ultimately increase when suitable measures are taken.

In recent times, ML algorithms have been widely used for the spatial prediction of several natural hazards such as flooding, landslides [16], wildfires [17], etc. Several researchers throughout the world have carried out GES mapping by using statistical as well as ML algorithms. Some of the widely used statistical methods to predict GES mapping are frequency ratio [7], logistic regression [18], weight of evidence (WoE) [19], index of entropy (IoE) [5], etc. Besides statistical methods, different ML algorithms have also been widely used to predict GES mapping such as artificial neural network (ANN) [20], support vector machine (SVM) [20], random forest (RF) (Hosseinalizadeh et al. 2019), multi-layer perception (MLPC) approaches [21], classification and regression tree (CART) [22], boosted regression tree (BRT) [7], particle swarm optimization (PSO) [23], multi-variate adaptive regression spline (MARS) [5], and maximum entropy [24]. Ensemble models have also been widely used for their novelties and capabilities in the comprehensive analysis of GES mapping [25]. Ensemble models are applied for high precision and predictive analysis of any kind of natural hazard susceptibility mapping. In other words, the presentation of an ML model is significantly enhanced by using an ensemble model. Along with machine learning models, different ensemble models have also been used for gully erosion modeling [20].

In very recent times, the deep neural learning network (DLNN) is a striking ML algorithm and has been widely used by several research groups. This method was proposed for the first time in 2006 and includes different key features of ML as well as artificial intelligence (AI). The DLNN algorithm consists of fully convolutional neural networks (CNNs), deep belief networks (DBNs), stacked auto-encoder (SAE) networks, etc. [26]. In addition to this, the Adaptive moment estimation (Adam) and Rectified Linear Unit (ReLU) algorithms were used for training and activation purposes in every learning unit of a DLNN model [27]. Generally, the DLNN algorithm has been used in different fields such as feature extraction and transformation through supervised and unsupervised processes, recognition of patterns, and classification [28]. On the other hand, the particle swarm optimization (PSO) algorithm is an extended part of AI and an amalgamation of the conventional ML techniques. The PSO algorithm is based on swarm intelligence, and it is straightforward with efficient universal optimization techniques [26]. PSO is used for the feature selection of a dataset through optimization techniques.

Deep learning (DL) and traditional ML algorithms have some basic differences, namely that the DL algorithm needs a big data size to perform and analyze successfully, and in the case of ML algorithms, they are performed in a certain way according to established rules. The DL algorithm requires a lot more matrix operation functions than the ML algorithm does to perform well [29]. In the case of the problem-solving method, the DL algorithm is done through end-to-end problem solving, whereas in the case of ML, it breaks down into multiple sub-problems. Therefore, the DL algorithm is much better than the traditional ML algorithm for mapping the GES zone. Thus, the greatest advantage of using the DLNN algorithm is that this model is capable of building a high-level feature from a raw dataset scientifically, and is also capable of delivering forecasting results using time series data. In addition to this, DLNN consists of a different topology than the general neural network of a single hidden layer; thus, more than one hidden layer is present in this algorithm. For this reason, in various research areas, the DL algorithm has better performance than the conventional ML algorithm [30]. In the case of PSO, it is also used to conquer the problems of local optima through feature selection methods. PSO determines the quality of a dataset’s features through a multi-objective fitness function [31]. As a result, the output layer of different hidden layers is optimized by the PSO algorithm to obtain more accurate predictions [32].

Therefore, the present research work has been carried out to predict GES mapping in Shirahan watershed, which is tremendously affected by water-induced gully erosion. To fulfill our research objective, we used thirteen suitable GECFs with a total of 132 gully head-cut points (each for gully and non-gully), splitting them into a 70/30 ratio for training and testing datasets. Furthermore, to creatively model the GES mapping, we used a DL as well as a conventional ML algorithm. In this study, we used DLNN, PSO, artificial neural network (ANN), and support vector machine (SVM) algorithms. According to several literature surveys on GES mapping and the best of our knowledge, it was noticed that the DLNN model has not been used in GES assessment so far; thus, this study was carried out to investigate the potential application of the DLNN model for GES mapping. In this study, an attempt was also made to use the PSO algorithm to optimize the parameters of the deep learning model (DLNN) in the training phase and to introduce a new approach of an ensemble of PSO and DLNN in GES modeling. Not only this, but a comparison was also made between the ensemble of PSO-DLNN and conventional ANN and SVM algorithms. Thus, the application of DL and the PSO-DLNN ensemble approach for GES mapping is the novelty in this research study, as the result of this approach improved the prediction accuracy compared with any single ML algorithm. Thereafter, all of the output results were validated through sensitivity (SST), specificity (SPF), positive predictive values (PPV), negative predictive values (NPV), receiver operating characteristic-area under the curve (ROC-AUC), likelihood ratio, F-measures, and maximum probability of correct decision (MPCD) statistical analyses. Thus, the DL and PSO-DLNN ensemble methods can help to forecast and control the creation and development of gullies in Shirahan watershed, Iran.

## 2. Materials and Methods

### 2.1. Description of the Study Area

**Figure 1:**
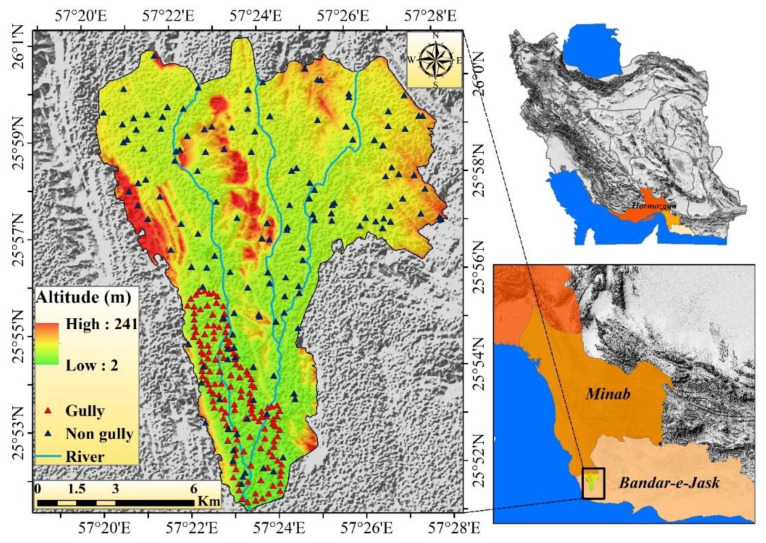
Location of the study area in Iran.

**Figure 2:**
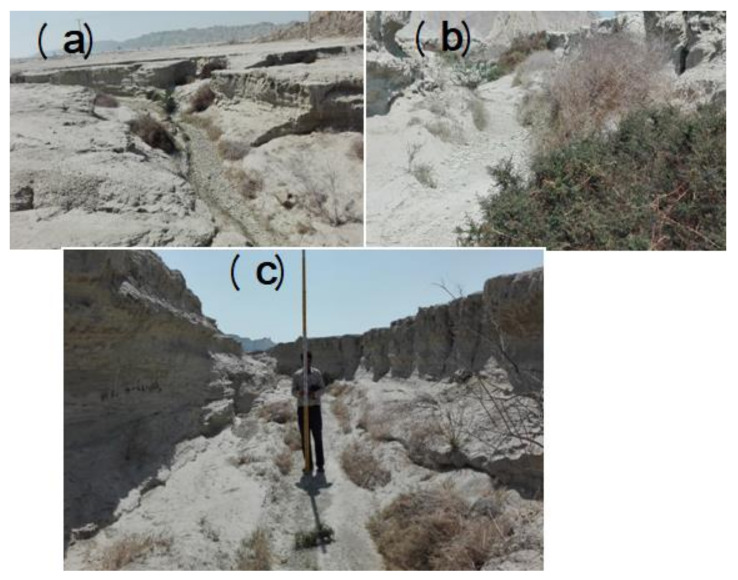
Images of gully erosion in Shirahan watershed: (**a**) head-cut gully; (**b**) gully erosion in salt land area; (**c**) measurement of gully morphometric properties.

Shirahan watershed is located at a longtitude of 20° 57′ to 28° 57′ and a latitude of 51° 25′ to 51° 26′, in the central part of Hormozgan Province and to the south of Bandar-e Jask city (Figure 1). The area of the watershed is 138 km^2^, the minimum height of the area is 2 m, and the maximum height is 214 m above sea level. According to statistics recorded at Jask Synoptic Station over a period of 28 years (1989–2017), precipitation in this region is very heavy, and more than 50% of it occurs in winter. According to the information of the above station, the average annual rainfall is 116.75 mm, the maximum annual rainfall is 320 mm, and the minimum is 27 mm. The climate of the region is hot and dry according to the Ambregeh method and hot/dry based on the Domarten method. Soil texture is generally silt/loam and loam. In this area, the percentage of clay increases with increasing depth. However, changes in the percentage of sand and silt do not follow a specific trend and have high fluctuations. In this area, the horizon of 75–75 cm has the highest degree of salinity (Table 1). Pictures of ditch erosion are shown in Figure 2. To study the geometric features and physical and chemical properties of the soil, 20 ditches were sampled in the study area. Studies showed further expansion of ditch erosion in salt marshes, which are located in the plain type. The general plan of ditches is compound, and their cross-sectional shape is trapezoidal. The average depth of ditches is 2.7 m; the average width is 10.3 m. Laboratory studies were used to evaluate the soil characteristics of the gullies of Shirahan watershed. Meanwhile, soil samples were taken from the soil surface to a depth of 290 cm and sent to the laboratory of Bandar Abbas Agricultural and Natural Resources Research Center for soil testing. Laboratory results showed that the soil texture in the area up to the depth under study is loose. The physical and chemical properties of the soil at 6 different soil depths are shown for the ditches studied in Table 1. Some field photographs of a gully in the present study area of Shirahan watershed are shown in Figure 2.

### 2.2. Methodology

**Figure 3:**
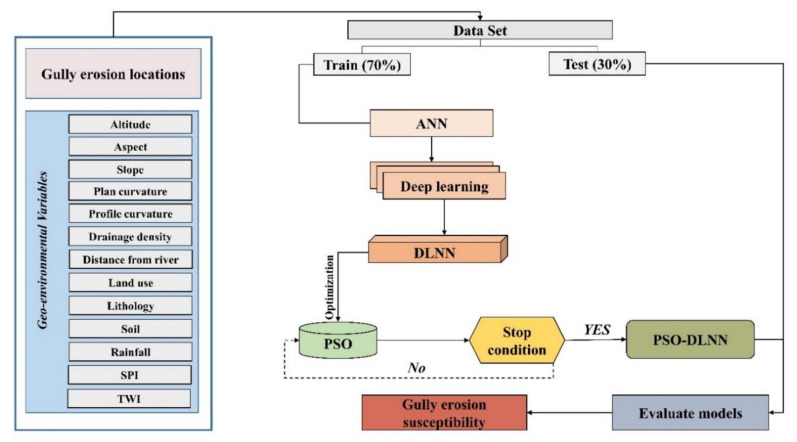
Methodological flowchart of particle swarm optimization (PSO)-deep learning neural network (DLNN) in gully erosion susceptibility.

The methodological approach used in this research work is discussed in the following section, and the respective flowchart is presented in Figure 3.

Firstly, a gully erosion inventory map was prepared based on the 132 gully head-cut points (with gully and non-gully for each). These gully head-cut points were identified based on field visits and information from the Administration of Natural Resources of Hormozgan Province. Along with this, the non-gully points were randomly selected throughout the basin area with the help of the geographic information system (GIS) environment. Besides this, a total of thirteen (13) of gully erosion conditioning factors (GECFs), i.e., target variables, were considered for modeling GES based on the local topographical and climatological factors in association with several literature studies. These GECFs are altitude, aspect, slope, plan curvature, profile curvature, drainage density (DD), distance from a river, land use, lithology, soil, rainfall, stream power index (SPI), and topographic wetness index (TWI). Thereafter, multi-collinearity analysis of variance inflation factor (VIF) and tolerance (TOL) techniques were used among different GECFs to determine the linear relationship among the variables. Afterwards, modeling of GES was done by using SVM, ANN, and DLNN machine learning (ML) algorithms, and a novel ensemble of PSO-DLNN. Lastly, the results of the several GES models were validated through a ROC curve analysis to assess their accuracy.

The methodology of the present research work was carried out to solve classification problems using the aforementioned ML and DL algorithms for prediction GES mapping. Besides this, the several target variables used in this study are a combination of logical, discrete, and continuous variables. During the processing of all of these variables’ data, it was recognized among the variables whether each one was a logical, discrete, or continuous one, in the SPSS 25 statistical software designed by International Business Machines (IBM), New York, USA. In this study, we also analyze affected areas of gully erosion susceptible zone, by using the presence of gully head-cut points, and we also compute the GES zones based on the gully/non-gully head-cut points along with several conditioning factors for sustainable management of the gully-affected areas.

### 2.3. Dataset Preparation for Spatial Modeling

In this study, a gully erosion inventory map (Figure 1) was prepared based on field visits and information from the Administration of Natural Resources of Hormozgan Province, which resulted in a total of 132 gully points. To determine the non-gully points, GIS software was used and 132 points were randomly selected. The digital elevation model (DEM) map was obtained with a pixel size of 12.5 m from the Advance Land Observatin Satellite/Phased Array type L-band Synthetic Aperture Radar (ALOSPALSAR) sensor. The topographical factors such as slope map, direction curve, plan curvature, and profile curvature were prepared based on DEM in the GIS environment. The map of the distance from a river based on the Euclidean extension was obtained in GIS software. A drainage density map was prepared using a line density extension. SAGAGIS software was used to map TWI and SPI. The soil type map of the region was obtained based on the map prepared by the Administration of Natural Resources of Hormozgan Province. The lithological map was prepared based on the geological map of 1:100,000 of the country’s mapping organization. Land use maps were prepared based on Landsat satellite images and Operational Land Imager (OLI) measurement, using the maximum probability algorithm in the ENVI software environment. The precipitation map of the constituency was prepared from the statistics of 4 climatological factors in the constituency over a period of 28 years (1989–2017) and based on the inverse distance weighting (IDW) interpolation method. Details about the data sources used in this research work are presented in Table 2.

**Figure 4:**
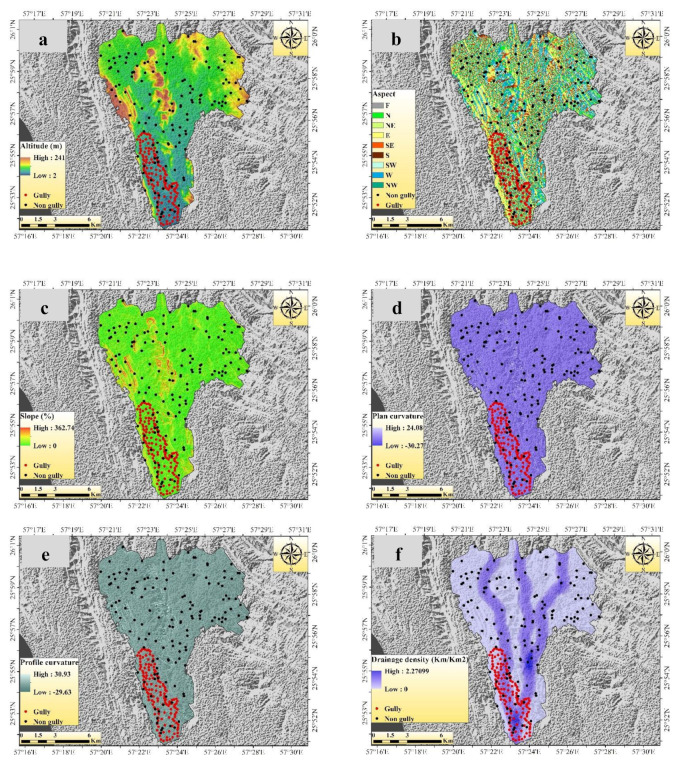

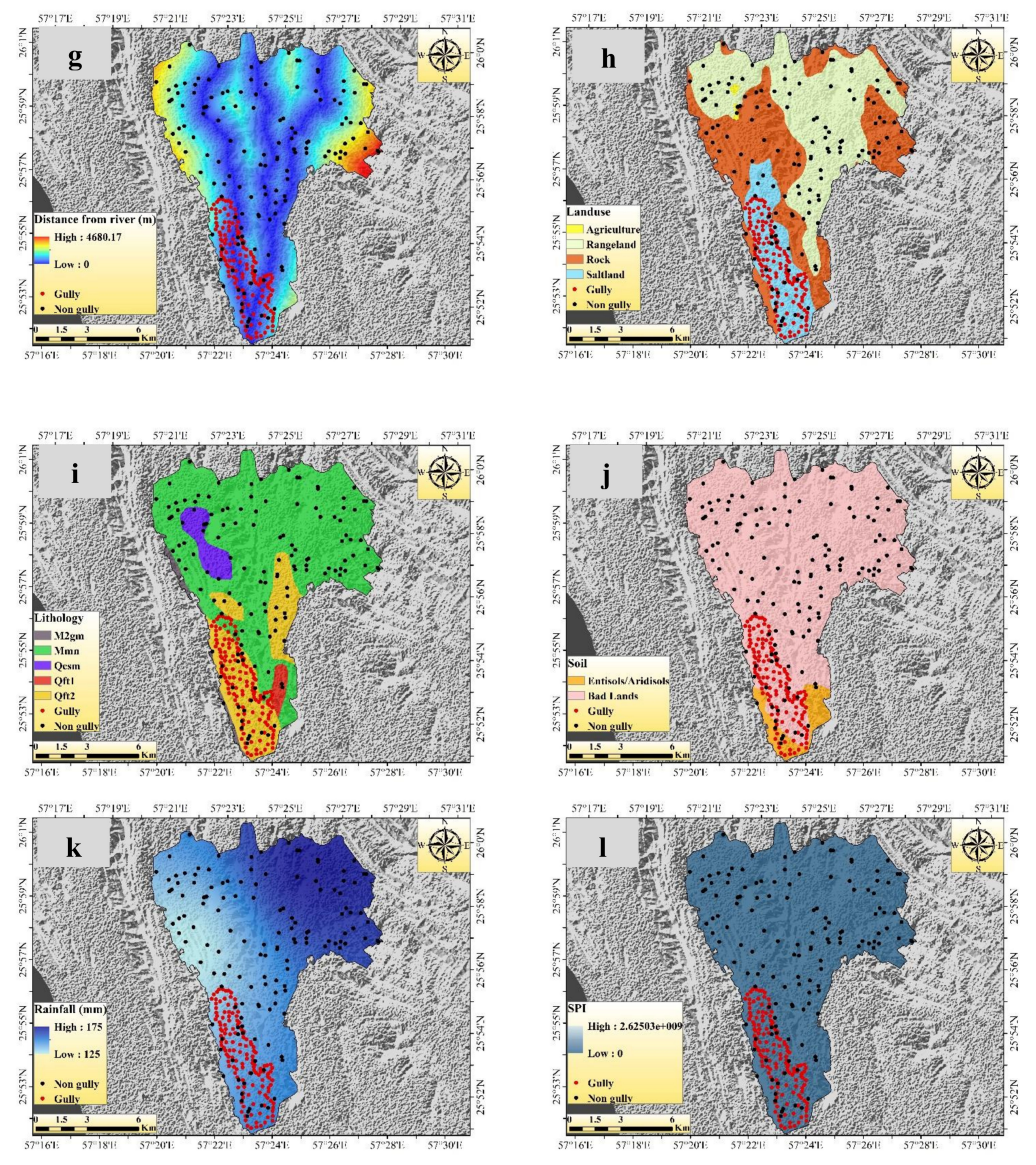

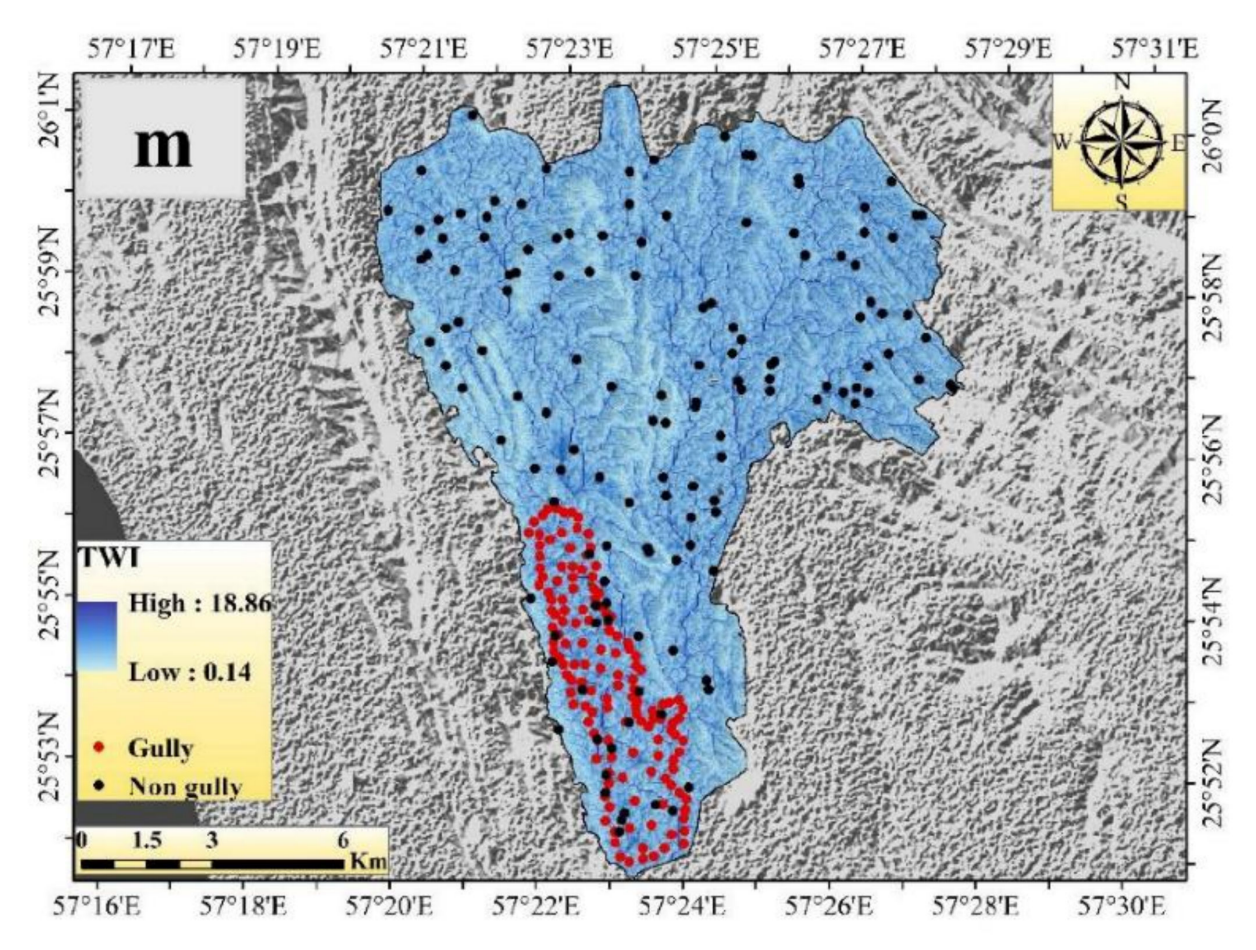
Gully erosion conditioning factors: (**a**) altitude, (**b**) slope, (**c**) aspect, (**d**) plan curvature, (**e**) profile curvature, (**f**) drainage density, (**g**) distance from a river, (**h**) land use, (**i**) soil, (**j**) lithology, (**k**) rainfall, (**l**) stream power index (SPI), and (**m**) topographic wetness index (TWI).

A total of 13 GECFs were selected for GES mapping in this research work, namely, altitude, aspect, slope, plan curvature, profile curvature, drainage density (DD), distance from a river, land use, lithology, soil, rainfall, stream power index (SPI), and topographic wetness index (TWI) (Figure 4a–m).

The altitude of the present study area ranges from 2 to 241 m (Figure 4a). Altitude is an important factor for the occurrence of gullies due to influences on rainfall-runoff processes, and it is largely employed in GES mapping [3]. Slope aspect indirectly affects the occurrence of gully erosion as it affects the reception of sunlight, vegetation cover, and humidity [33]. Here, the slope aspect map has nine classes, i.e., flat, N, NE, E, SE, S, SW, W, and NW (Figure 4b). Slope angle influences the pattern of runoff and infiltration rate. Therefore, depending on the slope, the erosional rate also varies from place to place, i.e., high slope areas have high erosion rates and vice versa. The slope map is shown in Figure 4c, and the value ranges from 0% to 362.74%. In a particular direction, the rate of gradient change is known as curvature, within which, plan and profile curvature generally represent the topographic characteristics of an area. The value of plan curvature ranges from −30.27 to 24.08 (Figure 4d) and profile curvature from −29.63 to 30.93 (Figure 4e). DD directly impacts occurrences of gully erosion. Horton’s (1932) following equation was used to calculate DD. In this study, the DD value ranges from 0 to 2.27 km/km^2^ (Figure 4f).

(1) $$DD=\frac{\sum_{i=1}^{n}S_{i}}{a}$$

where $\overset{n}{\underset{i=1}{\sum }}S_{i}$ is the length of drainage in km, and ‘*a*’ indicates the total area of the drainage basin in km^2^.

Distance from a river also influences occurrences of gully erosion as it greatly impacts the wetting capacity of surface area and associated erosional activities. The value of the distance from a river ranges from 0 to 4680.17 m (Figure 4g). The land use type of the area is very much responsible for the occurrence of gully erosion. Bare or less vegetated areas of the land surface are highly prone to gully erosion. In this study, four types of land use were recognized, i.e., agricultural land, rangeland, rock surface, and salt land (Figure 4h). The lithological factor of an area is highly responsible for erosional activities such as the development of a gully [34]. The present study area of Shirahan watershed consists of five types of lithological unit (Figure 4i). The soil map of the study area is shown in Figure 4j, and it is classified into two categories, i.e., entisols/aridisols and badlands. Rainfall is the most important factor for the formation of a gully and its development, mainly in the arid and semi-arid areas. High-intensity rainfall with short duration is the most devastating for gullies. Here, 28 years of rainfall data have been used to prepare a rainfall map (Figure 4k), and it ranges from 125 to 175 mm. SPI indicates the stream’s erosional capacity [25]. SPI value was calculated by using the following equations, and the value ranges from 0 to 2.625 in this research work (Figure 4l).

(2) SPI=As×tanβ

where *A_S_* represents the upslope contributing area, and β represents the slope angle.

TWI determines transport capacity along with flow velocity [7], and it is an essential factor for identifying gully erosion-prone areas [35]. The following equation was used to calculate TWI value, and it ranges from 0.14 to 18.86 (Figure 4m).

(3) $$TWI=In\left(\frac{A_{s}}{tan\beta }\right)$$

where *A_s_* represents the area of a catchment in m^2^, and *β* represents the gradient of the slope in radians.

### 2.4. Multi-Collinearity Analysis

Multi-collinearity analysis always gives the perfect outcome to evaluate the linear dependency of different geo-environmental factors in an ML model [15,36]. It is a statistical analysis and is able to find two variables of high correlation in a multiple regression study. Thus, it is very much essential to analyze the multi-collinearity of a model to obtain better results through removing the high multi-collinearity factors and minimizing the bias of the model [37]. Several researchers throughout the world have used multi-collinearity analysis in different fields such as GES mapping [21], floods [38], and landslide susceptibility mapping [39]. Multi-collinearity can be analyzed through variance inflation factor (VIF) and tolerance (TOL) [40]. As a general rule, if the TOL value is <0.10 or 0.20 and the VIF value is >5 or 10, then the result indicates high multi-collinearity among the variables [41]. The following equations were used to calculate TOL and VIF in a dataset:

(4) $$TOL=1-R_{j}^{2}$$

(5) $$VIF=\frac{1}{TOL}\;$$

where $R_{j}^{2}$ indicates the regression value of *j* on other different variables in a dataset.

### 2.5. Machine Learning Method Used in Modeling the Gully Erosion

#### 2.5.1. Support Vector Machine (SVM)

SVM is a very popular machine learning algorithm and was introduced by Vapnik and Chervonenkis in 1963. Several researchers throughout the world have used this machine learning classifier in the field of predicting different natural hazards such as in GES mapping [42], landslide prediction [43], flood susceptibility mapping [44], etc. SVM is implemented to solve regression analysis and multi-faceted classifier problems [45]. Vapnik [46] stated that SVM is based on the principle of structural risk minimization and statistical learning, and it is a supervised machine learning model. SVM is very much effective to reduce the error of the complexity of a linear computation and model overfitting [47]. Two types of statistically induced problems are engaged in SVM modeling. The first one is linear separating of the hyperplane by using statistical data, and the second one is converting non-linear data into linearly separable data [48]. Generally, the data processing in SVM of a non-linear relationship is done through the kernel function [49]. In addition to this, two classes can be discretely generated in SVM modeling by an optimal hyperplane, in which one class indicated above the hyperplane is assigned as 1 and the other one, located below the hyperplane, is assigned as 0, i.e., in this case gully erosion and non-gully erosion, respectively [50]. SVM has been developed for regression estimation, particularly paying attention to the solution of inverse problems. The novelty of the SVM model is that it has attempted to relocate the idea through kernel techniques for working out the inner products of unsupervised learning. Besides this, it can also be applied for singular components where the distribution of data is not well-defined. Therefore, a large class of functions can be applied for non-linearity mapping with high feature space by using this kernel trick. The hyperplane in an SVM can be calculated by using following equations:

(6) $$Min\overset{n}{\underset{i=1}{\sum }}\varphi_{i}-\frac{1}{2}\overset{n}{\underset{i=1}{\sum }}\overset{n}{\underset{j=1}{\sum }}\varphi_{i}\varphi_{j}y_{i}y_{j}\left(x_{i},x_{j}\right)\;\;\;\;\;\;\;$$

subject to

(7) $$Min\overset{n}{\underset{i=1}{\sum }}\varphi_{i}y_{j}=0and0\leq \alpha_{i}\leq D\;$$

where *x* = *x_i_*, *i* = 1, 2,… *n* are input variables of the vector; *y = y_i_*, *j = 1, 2,…n* are output variables of the vector, and $\varphi_{i}$ represents Lagrange multipliers.

Finally, the decision function of SVM can be classified as

(8) $$f\left(x\right)=sgn\left(\overset{n}{\underset{i=j}{\sum }}y_{i}\varphi_{i}K\left(x_{i},x_{j}\right)+a\right)$$

where a represents a bias, which indicates the linear distance of the hyperplane from the origin, $K\left(x_{i},x_{j}\right)\;$represent kernel functions, i.e., polynomial (POL) and radial basis function (RBF), and these can be expressed as follows [51]:

(9) $$K_{POL}\left(x_{i},x_{j}\right)={\left(\left(x*y\right)+1\right)}^{d}\;\;$$

(10) $$K_{RBF}\left(x_{i},x_{j}\right)=e^{-y{\left|\left|x-x_{i}\right|\right|}^{2}\;}$$

#### 2.5.2. Artificial Neural Network (ANN)

**Figure 5:**
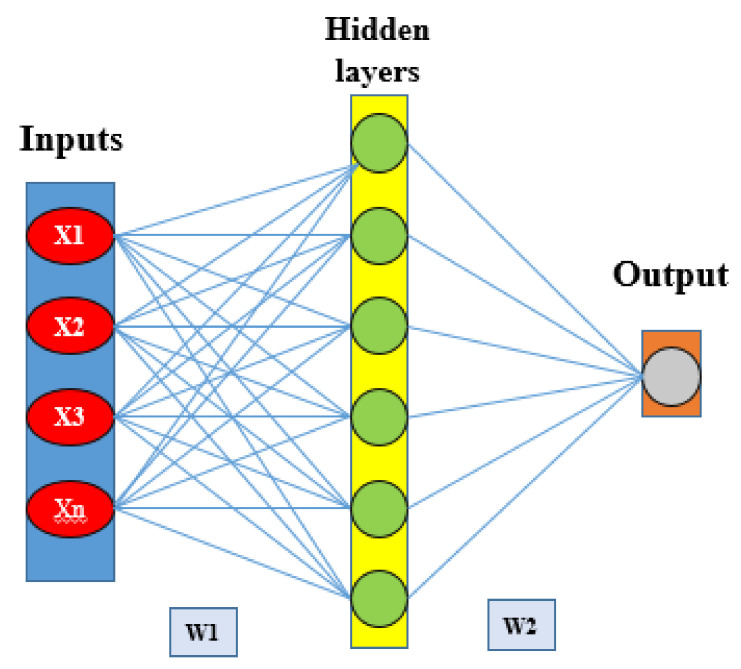
Schematic of feed-forward artificial neural network.

ANN is a popular machine learning algorithm that simulates the neural networks of a human brain and can work in a specific way [52,53]. It is used to analyze and predict non-linear statistical datasets by using different algorithms [54]. ANN has been widely used in pattern recognition and classification studies [55]. Therefore, classifications of the landscape in different ordinal areas of the GES zone are treated as a classification problem. Different types of algorithms have been used in ANN modeling; among them, multi-layer perceptron (MLP) is the most popular, based on its outcome results and frequency of use by researchers [56]. To run and analyze ANN algorithms, some basic knowledge is needed to understand the structure of input data and the relationship between the variables [57]. The ANN model with the MLP algorithm consists of three layers, namely, the input layer, hidden layer, and output layer. A schematic diagram of the feed-forward artificial neural network model is shown in Figure 5. In this research work, the input layers are training points for the erosion of the gully and the various GECFs, which have finally been connected to the output layer. Input nodes help to predict and analyze the model structure through input and hidden layers and, ultimately, to evaluate the output layer result [58,59]. This output layer gives us the GES map. The output layer consists of Boolean values of 0 and 1, in which 0 represents non-gully erosion and 1 represents gully erosion. Feed-forward of the ANN algorithm model deals with three stages, namely, feed-forward of input data, calculation, and backpropagation of related errors and their adjustments [57].

The novelty of the ANN model is that it can learn the model through a non-linear and complex relationship. Thus, the model’s uniqueness is evaluated based on observation of the coherence of the network dynamics compared with the other models. It also has the ability of model generalization and can predict unseen data within the model through understanding the hidden relationship.

The ANN algorithms were elaborated using the following equations by Hagan et al. (1996):

(11) $$net_{j}^{l}\left(t\right)=\overset{p}{\underset{i=o}{\sum }}(y_{i}^{i-1}\left(t\right)w_{ji}^{l}\left(t\right))\;\;\;\;\;\;$$

The net input of the *j*th neuron of layer *l* and *i*iteration

(12) $$y_{j}^{l}\left(t\right)=f(net_{j}^{\left(l\right)}\left(t\right)\;\;\;$$

(13) $$f\left(net\right)=\frac{1}{1+e^{\left(-net\right)}}$$

(14) $$e_{j}\left(t\right)=c_{j}\left(t\right)-a_{j}\left(t\right)\;\;\;\;\;\;\;\;\;\;\;\;\;\;\;\;\;\;\;\;\;\;\;\;\;$$

(15) $$\delta_{j}^{l}\left(t\right)=e_{j}^{l}\left(t\right)a_{j}\left(t\right)\left[1-a_{j}x\left(t\right)\right]\;$$

δ factor for the *j*th neuron in the *i*th output layer

(16) $$\delta_{j}^{l}\left(t\right)=y_{j}^{l}\left(t\right)\left[1-y_{j}\left(t\right)\right]\sum \delta_{j}^{l}\left(t\right)w_{kj}^{\left(l+1\right)}\left(t\right)$$

δ factor for the *j*th neuron in the *i*th hidden layer

(17) $$w_{ji}^{l}\left(t+1\right)=w_{ji}^{l}\left(t\right)+\alpha \left[w_{ji}^{l}\left(t\right)-w_{ji}^{l}\left(t-1\right)\right]+n\delta_{j}^{\left(l\right)}\left(t\right)y_{j}^{\left(l-1\right)}\left(t\right)\;$$

where α is the momentum rate and n is the learning rate.

#### 2.5.3. Deep Learning Neural Network (DLNN)

**Figure 6:**
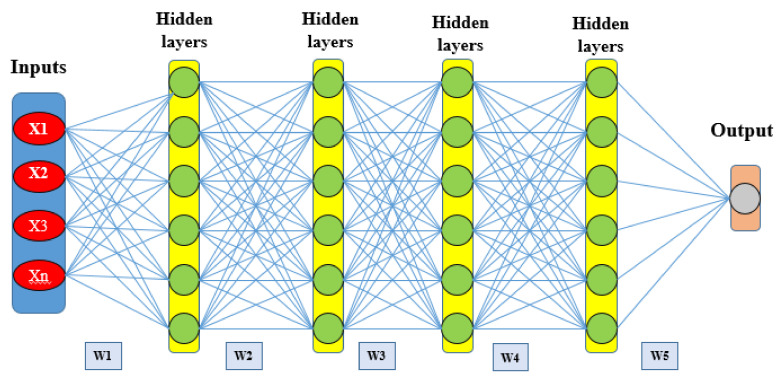
Schematic of deep learning neural network.

DLNN is a well-accepted machine learning model among research groups throughout the world. This ML model has a prominent advantage in appropriately constructing a high-level feature by using the raw dataset [27]. DLNN consists of three layers, i.e., an input layer, several hidden layers, and resulting in an output layer [60]. The speculative configuration of the DLNN model used for GES mapping in this research work is shown in Figure 6. The general structure of the DLNN model is to run in such a way that the input layer receives signals that are different GECFs, this information is processed and analyzed in several hidden layers, and finally, the output model’s result is presented in the last layer, i.e., the output layer. The output layer has two possible labels, i.e., the first one is a negative label (non-gully erosion) and the second one is a positive label (gully erosion). These classification results are obtained from the last hidden layer and shown in the output layer [61].

DLNN has some specific compensations over the traditional ML algorithm, and thus, in the field of prediction analysis, the use of the DLNN model has been given much more emphasis. Therefore, DLNN has showed some novel performances over the other ML models, namely, maximum utilization of unstructured data through relevant insights to understand the training dataset, being robust enough to recognize the novel data, and being able to develop additional learning models through adding more layers into the neural network system.

According to Kim (2017), the following mathematical equation is used in a DLNN machine learning model:

(18) $$h\left(x\right)=\left\{\begin{array}{c} x\;if\;x>0\\ 0\;if\;x\leq 0 \end{array}\right.=\max\left(0,x\right)\;$$

where x represents the input signal, and h indicates the activation function.

Based on the ReLU activation function, this can be as expressed as follows:

(19) $$h^{,}\left(x\right)=\left\{\begin{array}{c} 1\;if\;x>0\\ 0\;if\;x\leq 0 \end{array}\right.$$

The cost function is the difference between experiential and predicted class outputs. The loss function (*L*) of a cross-entropy used for pattern recognition and expressed as follows:

(20) $$L=-\frac{1}{N_{D}}\overset{N_{D}}{\underset{n=1}{\sum }}T1n\left(Y\right)+\left(1-T\right)1n\left(1-Y\right)\;\;$$

where $N_{D}$ represents the number of the training datasets, *T* indicates observed class outputs, and *Y* indicates predicted class outputs.

#### 2.5.4. Particle Swarm Optimization (PSO)

The algorithm of PSO is a meta-heuristic and was originally developed by an American social psychologist named Kennedy [62]. In our research work, we are faced with some non-linear problems, and to find the correct solution, the PSO method was developed and widely used. The PSO algorithm was used to locate the best possible food route for bird and fish intelligence. Here, birds are the particles and try to find a solution to the problem. Particles always try to find the best possible solution to a problem through *n*-dimensional space, in which *n* represents each problem’s different parameters [63]. Optimization of position and velocity is the basic principle of each particle.

Therefore, let us suppose that $x_{i}^{t}=\left(x_{i1}^{t},x_{i2}^{t},\;\ldots ,x_{in}^{t}\right)and\;v_{i}^{t}=\left(v_{i1}^{t},v_{i2}^{t},\;\ldots ,v_{in}^{t}\right)$ are the position and velocity of changing position designed for the *i*th particle in the *t*th iteration accordingly. The following equations are used for the *i*th particle’s position and velocity in the *(t+1)*th iteration:

(21) $$v_{i}^{t+1}=\omega .v_{i}^{t}+c_{1}.r_{1}.\left(p_{i}^{t}-x_{i}^{t}\right)+c_{2}.r_{2}.\left(g_{i}^{t}-x_{i}^{t}\right)\;with-v_{max}\leq v_{i}^{t+1}\leq v_{max}\;$$

(22) $$x_{i}^{t+1}=\left(x_{i}^{t}+v_{i}^{t+1}\right)$$

where $x_{i}^{t}$ represents the previous *i*th position; $p_{i}^{t}$ represents the optimal found position; $g_{i}^{t}$ represents the particle’s best position;$\;r_{1}\;$and $r_{2}$ represent random numbers of either 0 or 1; ω is weights of inertia; $c_{1}$ is a coefficient; and $c_{2}$ represents the social coefficient. There are numerous methods for particle weight assignment [64,65]; among them, standard 2011 PSO has been widely used and can be calculated by the following equation:

(23) $$\omega =\frac{1}{2\;In\;2}and\;c_{1}=c_{2}=0.5+In\;2$$

**Figure 7:**
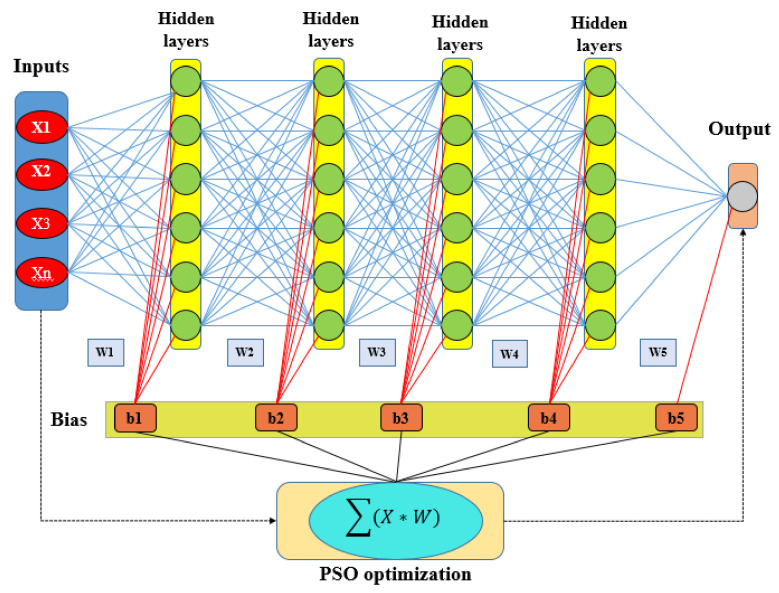
Schematic of ensemble particle swarm optimization and deep learning neural network.

Therefore, it is believed that when the concentration of all particle swarms in a certain point and space has been achieved, the problem has been solved. The intelligence-based PSO algorithm has been widely used in high-efficiency swarm paralleling and optimization property. Using a multi-objective fitness function, PSO determines the quality of several features in a dataset. Finally, the ensemble structure of particle swarm optimization (PSO) and deep learning neural network (DLNN) is shown in Figure 7. Therefore, this ensemble method is the novel approach in this research study for GES mapping with high accuracy.

### 2.6. Methods of Validation and Accuracy Assessment

GES maps were prepared based on the prediction performance of the training and validation datasets by using different machine learning models. Therefore, it is necessary to evaluate the model performance to ascertain the validity of the results. In the present research work, statistical indices along with the area under the receiver operating characteristic (AUROC) curve were used to predict the accuracy of ML and ensemble models.

#### 2.6.1. Statistical Indices

In this study, sensitivity (SST), specificity (SPF), positive predictive values (PPV), and negative predictive values (NPV) were used to evaluate the predictive results. Four types of possible consequences were used to analyze these statistical indices, namely, true positive (TP), true negative (TN), false positive (FP), and false negative (FN). TP is when gully pixels are correctly classified as a gully, and FP is when gully pixels are incorrectly classified as a gully. On the other hand, if gully pixels are correctly or incorrectly classified as non-gully, then they are TN and FN, respectively [36]. If higher values are found among these statistical indices then the model gives better results and vice versa [22]. The following equations were used to calculate the value of these four statistical indices:

(24) $$PPV=\frac{TP}{FP+TP}$$

(25) $$NPV=\frac{TP}{TP+FN}$$

(26) $$SST=\frac{TP}{TP+FN}$$

(27) $$SPF=\frac{TN}{FP+TN}$$

#### 2.6.2. ROC Curve

ROC curve is one of the most widely used tools for analyzing the performance validation of the ML model. ROC curve has two dimensions, i.e., events and non-events phenomena [66]. This curve is plotted on ‘X’ and ‘Y’ co-ordinates, known as sensitivity and 1-specificity, respectively, and represents true positive and false positive. The optimum value in both cases, i.e., in sensitivity (detected gullies) and specificity (detected non-gullies), is 1 [3]. The value of ROC-AUC ranges from 0.5 to 1, in which 0.5 indicates poor performance and 1 indicates very good performance. Beside this, in a proper way it can be classified into five classes, i.e., poor (0.5–0.6), moderate (0.6–0.7), good (0.7–0.8), very good (0.8–0.9), and excellent (0.9–1) [67]. The following equation was used to compute the ROC-AUC:

(28) $$S_{AUC}=\overset{n}{\underset{k=1}{\sum }}\left(X_{k+1}-X_{k}\right)\left(S_{k}+1-S_{k+1}-\frac{S_{k}}{2}\right)\;\;\;$$

where $S_{AUC}$ indicates area under the curve, $X_{k}$ indicates 1-specificity, and $S_{k}\;$indicates the sensitivity of the receiver operating characteristic (ROC) curve.

Besides the above validation methods, here we also used Likelihood Ratio (LR), F-measure, and Maximum Probability of Correct Decision (MPCD) analyses to better understand the accuracy assessment of the result. In this study, the LR model is the relationship between the distribution of gully head-cut points and related GECFs. Therefore, the LR model emphasized the ratio of the probability of events and non-events phenomena of the gully occurrences. In this method, if the ratio is higher than 1, there is a high relationship among the gully erosion and associated factors. On the other hand, if the ratio is less than 1, a low relationship is found between the gully erosion and associated factors. Thus, the linear relationship of LR can be expressed as follows:

(29) GESI=∑Fr 

where *GESI* represents the gully erosion susceptibility index, and *Fr* represents the rating of several factors’ range.

*F-measure* is a popular validation method in the field of classification and information retrieval communities. *F-measure* balances between precision and recall. The following equation was used to calculate the *F-measure* in this study:

(30) F−measure=2×TP/2×TP+FP+FN

In a classification performance, MPCD is a probabilistic-based measure. It is a sensitive method for recognition of class rather than just to estimate the proportion of guesses. The following equation was used to calculate the MPCD:

(31) $$MPCD=\left(1-\alpha \right)\left(1-\beta \right)$$

where α is $\frac{FP}{FP+TN}$ and β is $\frac{FN}{FN+TP}$.

## 3. Results

### 3.1. Multi-Collinearity Analysis

Maintaining the given VIF and TOL limits, 13 gully erosion conditioning parameters were selected for gully erosion modeling. The co-linear factors (i.e., distance from a road, geomorphology, and bulk density) were excluded from this analysis. The three factors of distance from a road (TOL 0.028 and VIF 35.65), geomorphology (TOL 0.032 and VIF 31.63), and bulk density (TOL 0.022 and VIF 45.23) are associated with co-linearity problems. The range of VIF for the selected parameters is 1.06 to 3.04. In the case of TOL, the range of variation among the selected conditioning factors is 0.33 to 0.94 (Table 3). Among the 13 GECFs, altitude has the highest VIF value of 3.04 and the lowest TOL value of 0.33. On the other hand, the aspect factor has the highest TOL value of 0.94 and the lowest VIF value of 1.06. Therefore, this indicates that no multi-collinearity has been found between the thirteen conditioning factors of gully erosion used in this study.

### 3.2. Gully Erosion Susceptibility Modeling

**Figure 8:**
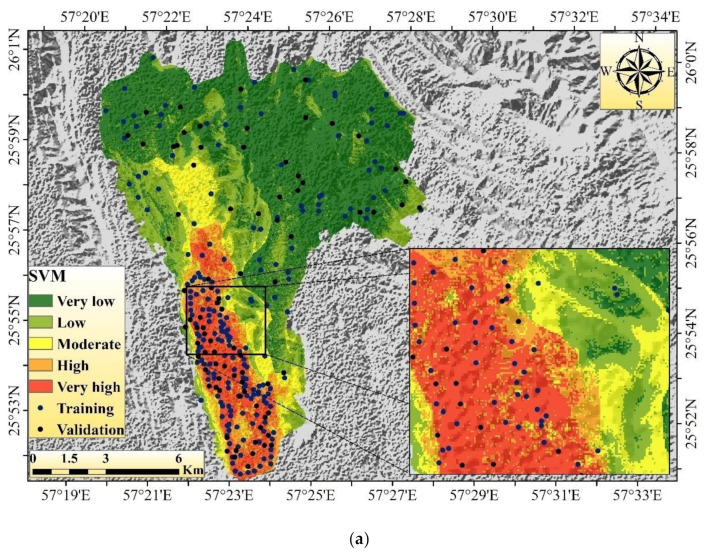

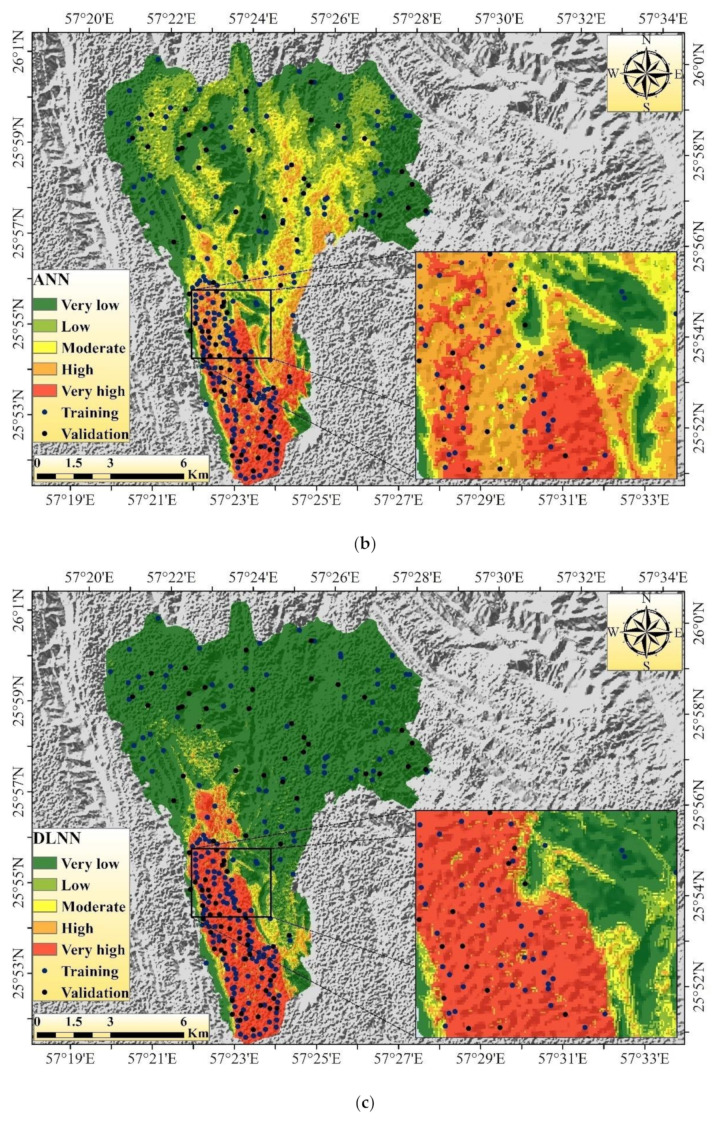

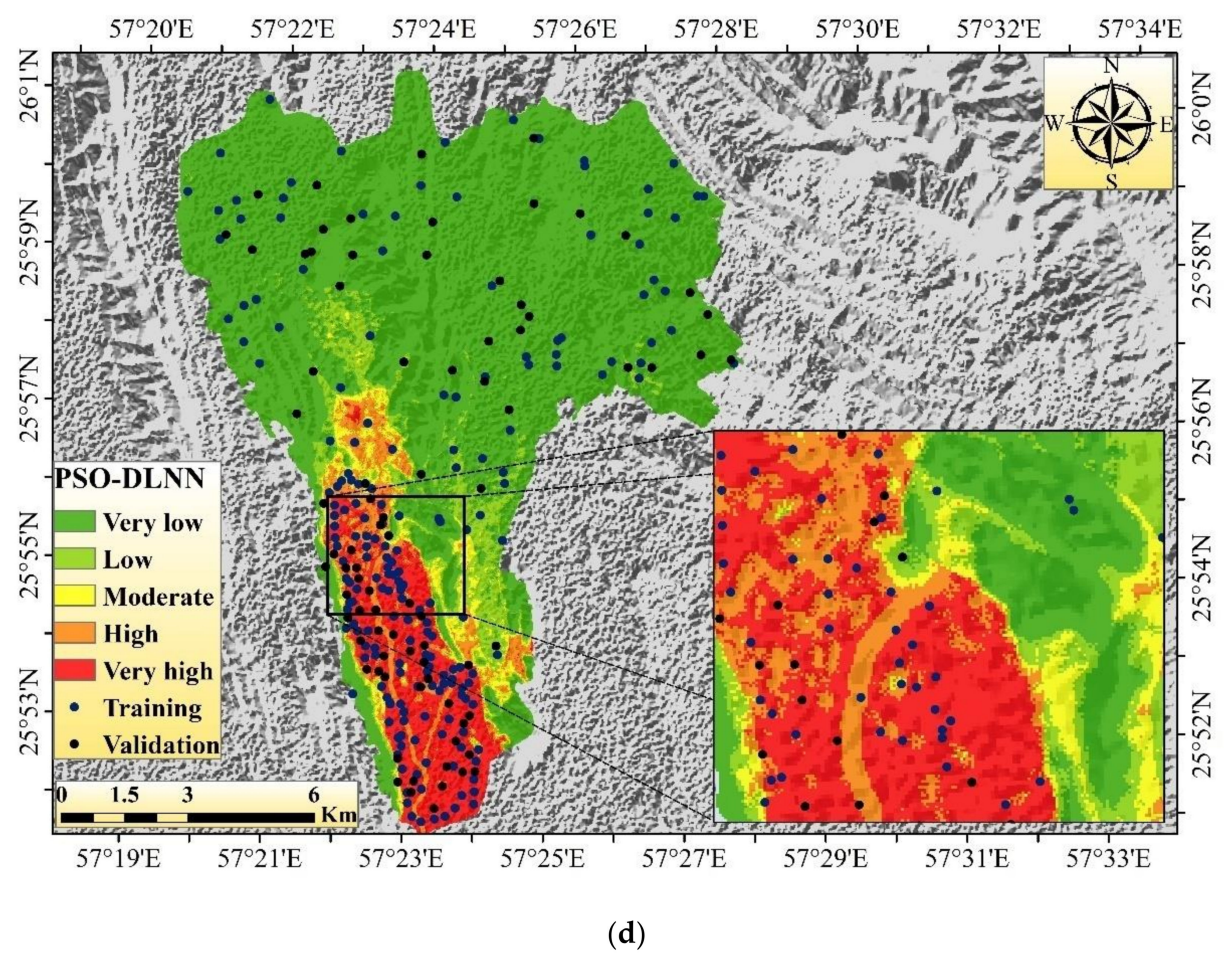
Head-cut gully erosion map using the four models: (**a**) SVM; (**b**) ANN; (**c**) DLNN; (**d**) PSO-DLNN.

In the SVM model, the very low GES areas are mainly concentrated in the eastern and northern portions of the region. The low GES areas are mainly found in the middle and western parts of the region. The moderate susceptibility areas are mainly concentrated in the middle and southern parts of the region (Figure 8a). The very high and high GES areas are mainly found in the southern portion of the watershed. The areal coverages of very low, low, moderate, high, and very high gully erosion susceptibility areas in the SVM model are 65.86 (52.08%), 28.92 (22.87%), 10.7 (8.46%), 8.0 (6.33%), and 12.97 km^2^ (10.26%), respectively (Table 4).

In ANN, the areal coverages for very low, low, moderate, high, and very high gully erosion susceptibility areas are 55.76 (44.10%), 26.85 (21.23%), 16.85 (13.33%), 13.48 (10.66%), and 13.51 km^2^ (10.68%), respectively. According to the GES map of the ANN model, the largest portion of the area is occupied by very low (44.10%) to low (21.23%) susceptibility classes, while very high (10.68%), high (10.66%), and moderate (13.33%) susceptibility classes cover the rest of the studied region. In this model, the very high, high, and moderate susceptibility areas are mainly concentrated in the southern, middle, and eastern portions of the watershed (Figure 8b). The rest of the portion of this watershed is associated with very low to low GES zones.

In the case of DLNN, the areal coverages for very low, low, moderate, high, and very high gully erosion susceptibility zones are 96.34 (76.19%), 5.85 (4.63%), 2.73 (2.16%), 3.17 (2.51%), and 18.36 km^2^ (14.52%), respectively. According to the GES map of the DLNN model, the largest portion of the area is occupied by very low (76.19%) to low (28.73%) susceptibility classes, while very high (14.52%), high (2.51%), and moderate (2.16%) susceptibility classes occupy the rest of the studied region. In this model, the very high to moderate susceptibility areas are mainly concentrated in the southern and middle portions of the watershed, and the rest of the portions are associated with very low to low susceptibility zones (Figure 8c).

In the PSO-DLNN model, the areal coverages of low, low, moderate, high, and very high gully erosion susceptibility zones are 94.58 (74.80%), 8.15 (6.45%), 4.03 (3.19%), 6.31 (4.99%), and 13.38 (10.58%) km^2^, respectively. According to the GES map of the PSO-DLNN model, the major portion of the area is occupied by very low (74.80%) to low (6.45%) susceptibility classes, while very high (10.58%), high (4.99%), and moderate (3.19%) susceptibility classes cover the rest of the studied region respectively. Very high, high, and moderate gully erosion susceptibility zones mainly occupy the southern portion of the watershed, and the rest of the portions are associated with very low to low susceptibility zones (Figure 8d).

### 3.3. Validation of the Models

**Figure 9:**
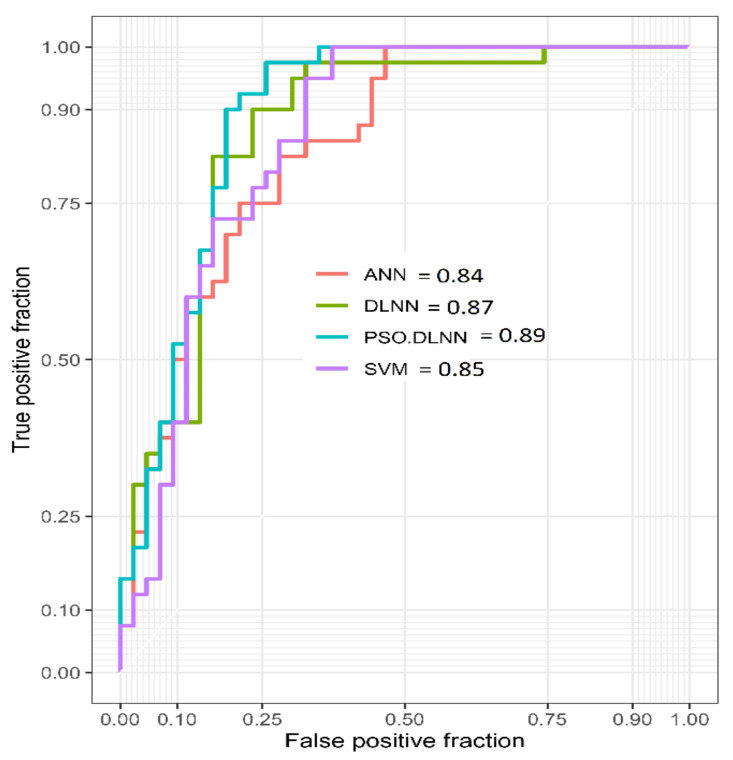
Receiver operating characteristic (ROC) curve analysis for four head-cut gully erosion models using the testing dataset.

PSO-DLNN is the most optimal model in this analysis and is associated with maximum accuracy. The AUC value from ROC considering the testing datasets of PSO-DLNN is 0.89, which is associated with superb accuracy. The rest of the models are also associated with optimal accuracy and have similar values to the PSO-DLNN model; the AUC values from ROC of DLNN, SVM, and ANN for testing datasets are 0.87, 0.85, and 0.84, respectively (Figure 9). Apart from this, various statistical indices were considered for estimating the optimal capacity of all the models for GES modeling. The values of sensitivity in PSO-DLNN, DLNN, SVM, and ANN for training datasets are 0.98, 0.95, 0.99, and 0.99, respectively. The same values for the validation datasets in PSO-DLNN, DLNN, SVM, and ANN are 0.95, 0.90, 0.82, and 0.95, respectively. The values of specificity for the training datasets in PSO-DLNN, DLNN, SVM, and ANN are 0.85, 0.82, 0.86, and 0.87, respectively. In the case of validation datasets, the values of specificity in PSO-DLNN, DLNN, SVM, and ANN are 0.74, 0.74, 0.69, and 0.67, respectively. The values of PPV in the case of training datasets in PSO-DLNN, DLNN, SVM, and ANN are 0.87, 0.85, 0.88, and 0.89, respectively. When we consider the validation datasets, the values of PPV in PSO-DLNN, DLNN, SVM, and ANN are 0.77, 0.77, 0.71, and 0.73 (Table 5). In PSO-DLNN, DLNN, SVM, and ANN models, the values of NPV for the training datasets are 0.97, 0.94, 0.99, and 0.99, respectively. In the case of validation datasets, the values of NPV in PSO-DLNN, DLNN, SVM, and ANN are 0.94, 0.89, 0.81, and 0.93, respectively. The F-measure values in validation datasets for PSO-DLNN, DLNN, SVM, and ANN models are 0.66, 0.635, 0.63, and 0.64, respectively.

**Figure 10:**
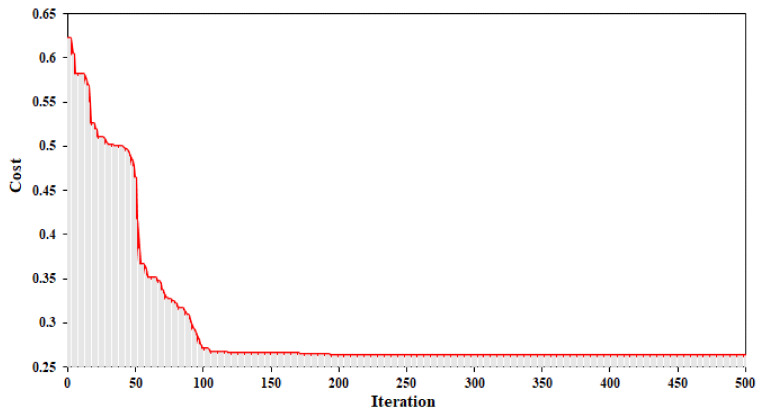
Convergence graph of the objective cost function (MSE) in the PSO-DLNN model.

Details about DLNN and its associated parameters are shown in Table 6. Details about the combination of PSO and DLNN and its associated parameters are shown in Table 7. The values of population, iteration, phi, phi1, phi2, W, C1, C2, and best cost are 50, 500, 4.1, 2.05, 2.05, 0.73, 1.49, 1.49, and 0.26. The objective cost function of the PSO-DLNN model is shown in Figure 10.

### 3.4. Variable Importance

The conditioning factor for GES modeling for this region was selected considering the different kinds found in the literature. The most important parameters for the creation and development of gullies in this region are land use, altitude, lithology, rainfall, and distance from a road, etc. The relative importance of land use, altitude, lithology, rainfall, and distance from a road for the GES models are 100, 97.94, 59.51, 46.94, and 29.48, respectively. The rest of the factors (i.e., profile curvature, TWI, plan curvature, slope, soil, drainage density, SPI, and aspect) are associated with moderate to very low relative importance for GES. The relative importance of profile curvature, TWI, plan curvature, slope, soil, drainage density, SPI, and aspect for gullies are 16.22, 14.37, 11.31, 7.89, 7.1, 6.91, 5.12, and 0 (Table 8). Here, apart from the topographical and geohydrological characteristics, the impact of anthropogenic activities accelerates the rate of land degradation in the form of gullies.

## 4. Discussion

Land degradation through various forms of soil erosion can cause extensive damage, and it has an adverse impact on society and people’s livelihoods throughout the world [68]. There are various forms of erosion, i.e., sheet erosion, formation of rills, formation and development of gullies and ravines, etc. [69]. Of these, the formation and development of gullies and their associated erosion is the most destructive element of land degradation worldwide [2]. Although it is a natural process of erosion, this process can be greatly accelerated by anthropogenic activities and have a serious impact on the ecosystem [70]. With this type of erosion, agricultural activities have not only affected it but have also been associated with damage to manmade infrastructure. On the one hand, this erosion is responsible for removing the top soil, but on the other hand, it is responsible for the creation and accumulation of sediment in the lower catchment area [71]. The life span of the reservoir will cause serious damage to the sediment deposition resulting from this type of erosion [72,73].

Shirahan watershed in Iran has recently faced severe gully erosion, which is responsible for large-scale erosion and is the main barrier to sustainable land management practices. Therefore, identifying vulnerable regions with the most optimal model is very useful so that appropriate soil and water conservation measures can be put in place. For this purpose, we considered SVM, ANN, DLNN, and PSO-DLNN in order to estimate the GES of this region with the maximum possible accuracy and to suggest the most suitable model. The erosion of a gully is controlled by various causal factors, and we attempted to determine the importance of these factors for gulling. Apart from the topographic and hydrogeomorphic attributes, land use is the most important variable for gully erosion, which indicates the large anthropogenic impact on the development of gullies. Other factors, such as altitude, lithology, rainfall, and the distance from a river, are very influential too on gully erosion and promote gulling. The transformation of land use is a crucial element and is responsible for large-scale erosion [74]. Alterations in land use influence landscape ecology functions, with far-reaching implications for natural ecosystems and land reclamation [75]. The character and volume of the surface runoff may change directly with the changing pattern of land use in the region. From this perspective, the nature of erosion in the form of gullies can have a significant effect on the impact of rainfall and its associated runoff characteristics in a changing environment. This type of outcome is similar to some of the findings from the studies of a number of researchers in this diversified discipline. This finding has been highlighted by many other contributions in which morphological and geological properties are assigned as the determinants of the highest possible location of GES [24,76]. Other research outcomes suggest that environmental and hydrological parameters are very significant and responsible for gulling.

All the predicted models are associated with high accuracy, but PSO-DLNN is the most optimal, with the AUC of this model being 0.89. The efficiency of all predicted models is excellent, with the AUC values for DLNN, SVM, and ANN being 0.87, 0.85, and 0.84, respectively. Apart from this, considering various statistical indices, PSO-DLNN is the best model among the models used in this study. According to the PSO-DLNN model, 18.76% of the total area is associated with a moderate to very high susceptible area of gully erosion. The southern portion of this watershed is mainly associated with higher gully occurrences. The complex geohydrological characteristics of this region are favorable for large-scale erosion in the form of gullies.

A deep learning framework is associated with higher accuracy compared with conventional ANN and SVM ML methods. This model can handle a larger number of samples and even a large amount of big data, and can estimate the results with optimum accuracy. The traditional ML algorithm is not capable of handling this large a number of samples, and the outcome from this perspective is less optimal compared to the deep learning framework. Significant progress in DLNN-dependent deep learning (DL) systems has significantly increased the consistency of machine learning for various purposes. While the standardized features of multi-layer NNs are well-established, the main advantage of DL is its structured method of self-governing the training of DLNN layer organizations. The benefits of structured data and expertise descriptions were recognized before the recent increase in interest in DLNNs. This definition is widespread in the physical sciences where the proposed method is popular for both specific theoretical structures and complicated system implementations in practice.

First, PSO produces an arbitrary solution and then discovers accurate solutions with an incremental optimum fitness attribute. This type of methodology has already been used primarily for backpropagation (BP) genetic algorithms, due to the efficiency of simple installation, fast response, and accuracy of predictions. It also demonstrated dominance in the resolution of complex applications and was initially implemented in the context of DL. The best function of the PSO algorithm is to combine various particles that are interlinked to each other to achieve an optimum position. The same technique indicates the position, velocity, and highest accuracy of each particle, which are dictated by the basic concepts used to enhance the problem. Particularly in comparison to other optimization algorithms, the advantage of the PSO algorithm is that the PSO technique usually involves a quick and important search procedure, is easy to perform, and can find the globally optimal path that is closest to the concrete ideas.

## 5. Conclusions

It is necessary to choose the most efficient machine learning algorithm in order to decrease the inconsistencies associated with predicting gully erosion susceptibility. The main objectives in most cases of susceptibility modeling are to identify the optimal model according to its predictive capabilities. The identification of key parameters for the formation and development of gullies is necessary to estimate the susceptibility mapping of the spatial distribution of gully erosion. Therefore, to control damage in the future, it is important to make an appropriate selection of a model to manage areas that are prone to gully degradation. The primary objective of this research was to estimate the optimal model with maximum predictive capability. For this reason, various ML algorithms, i.e., ANN, SVM, DLNN, and PSO, were considered for estimating the GES zone with optimal capacity. PSO-DLNN is the best-fitted model and is associated with the highest AUC value (0.89). Here, all the datasets were randomly partitioned with a 70/30 ratio as training and validation datasets. Topographical, hydrological, and environmental factors were most dominant and were influential factors in susceptibility modeling. The role of land use in susceptibility modeling is more significant than that of any other component. Most of the region of this watershed is associated with very low to low susceptibility zones, while 15.57% of the area is associated with a very high susceptibility zone. This study region must take appropriate planning initiatives to reduce the level of vulnerability and to protect this precious resource. In future research, it would be desirable to develop the PSO-DLNN algorithm by incorporating some new components or to develop the same algorithm with slight modifications. This would be a great contribution to the research community as well as to society. Apart from this, the selection of inappropriate parameters can reduce the efficiency of the predicted models. Thus, the selection of the most appropriate variables for susceptibility modeling is one of the important tasks for researchers.

## Figures and Tables

**Table 1:** Physical and chemical properties of soil in the gullies of Shirahan watershed.

Features	Soil Depth (cm)					
	0–30	30–75	75–130	130–180	18–250	250–290
PH	8.06	7.59	8.19	7.69	8.32	7.38
EC (mmhos/cm)	2.26	34.6	2.23	33.9	2.4	33.2
Na (Meq/lit)	8.82	285	8.87	285	8.8	248
Ca + Mg (Meq/lit)	13.6	64.4	13.7	62.4	13.4	63.2
SAR	3.4	50.2	3.4	50.2	3.4	50.2
Clay (%)	24	26	26	27	29	28
Silt (%)	58	30	60	32	56	30
Sand (%)	18	44	14	41	15	42
Soil texture	Silt-Loam	Loam	Silt-Loam	Loam/clay-loam	Silty-Clay-Loam	Clay-Loam

**Table 2:** Details about the data sources of several factors used in this study.

Parameters	Data Source	Time (Year)	Spatial Resolution/Scale
Altitude, slope, aspect, profile curvature, plan curvature, drainage density (DD), distance from river, stream power index (SPI), topographic wetness index (TWI)	ALOS PALSAR DEM (Alaska Satellite Facility)	2012	12.5 m
Rainfall	Iran Meteorological Organization (IMO) (http://www.weather.ir/)	1989 to 2017	
Lithology	Geological Survey of Iran (GSI) (http://www.gsi.ir/)	2019	1:1,000,000
Land use	Landsat OLI 8 satellite image (USGS)	2019	30 m
Soil texture	Soil and Water Research Institute (http://www.iran.swri.com)	2019	1:1,000,000

**Table 3:** Multi-collinearity analysis to determine the linearity of the independent variables.

Variables	VIF	Tolerance
Altitude	3.04	0.33
Slope	1.34	0.75
Aspect	1.06	0.94
Plan curvature	1.83	0.55
Profile curvature	1.82	0.55
Distance from river	2.93	0.34
Drainage density	2.07	0.48
Rainfall	1.41	0.71
Land use	1.81	0.55
Lithology	2.07	0.48
Soil	1.11	0.90
SPI	1.58	0.63
TWI	1.94	0.52

**Table 4:** Areas of gully erosion susceptibility classes.

Models	Area	Susceptibility Class				
		Very Low	Low	Moderate	High	Very High
SVM	km^2^	65.86	28.92	10.7	8	12.97
	%	52.08	22.87	8.46	6.33	10.26
ANN	km^2^	55.76	26.85	16.85	13.48	13.51
	%	44.10	21.23	13.33	10.66	10.68
DLNN	km^2^	96.34	5.85	2.73	3.17	18.36
	%	76.19	4.63	2.16	2.51	14.52
PSO-DLNN	km^2^	94.58	8.15	4.03	6.31	13.38
	%	74.80	6.45	3.19	4.99	10.58

**Table 5:** Predictive capability of guully erosion susceptibility (GES) models using training and testing datasets.

Models	Stage	Parameters					
		Sensitivity	Specificity	PPV	NPV	AUC	F-Measure
SVM	Training	0.99	0.87	0.89	0.99	0.94	0.84
	Validation	0.95	0.67	0.73	0.93	0.85	0.63
ANN	Training	0.99	0.86	0.88	0.99	0.94	0.83
	Validation	0.82	0.69	0.71	0.81	0.84	0.64
DLNN	Training	0.95	0.82	0.85	0.94	0.91	0.82
	Validation	0.90	0.74	0.77	0.89	0.87	0.65
PSO-DLNN	Training	0.98	0.85	0.87	0.97	0.93	0.84
	Validation	0.95	0.74	0.77	0.94	0.89	0.66

**Table 6:** Results of optimal parameters in the DLNN model.

Parameters	Optimum
Input number of units	13
Output	2
Activation Function	ReLU
Activation	‘softmax’
Function	Sigmoid
reluLeak	0.01
eta	0.8
Hidden layer unit	3-3
Iteration	200

**Table 7:** Parameters used in PSO algorithms in combined DLNN.

Parameters	Number
Population	50
Iteration	500
phi	4.1
phi1	2.05
Phi2	2.05
W	0.73
C1	1.49
C2	1.49
Best Cost	0.26

**Table 8:** Variable importance analysis based on the PSO-DLNN model.

Variables	Importance
Altitude	97.94
Aspect	0
Slope	7.89
Plane curvature	11.31
Profile curvature	16.22
Drainage density	6.91
Distance from river	29.48
Land use	100
Lithology	59.51
Soil	7.1
Rainfall	46.94
SPI	5.12
TWI	14.37
